# PET imaging of brain inflammation during early epileptogenesis in a rat model of temporal lobe epilepsy

**DOI:** 10.1186/2191-219X-2-60

**Published:** 2012-11-08

**Authors:** Stefanie Dedeurwaerdere, Paul D Callaghan, Tien Pham, Gita L Rahardjo, Halima Amhaoul, Paula Berghofer, Mitchell Quinlivan, Filomena Mattner, Christian Loc'h, Andrew Katsifis, Marie-Claude Grégoire

**Affiliations:** 1 Department of Translational Neuroscience, University of Antwerp, FGEN CDE T4.20, Universiteitsplein 1, Wilrijk, Antwerp, 2610, Belgium; 2 LifeSciences, ANSTO, Locked Bag, Kirrawee DC, NSW, 2232, Australia; 3 Department of PET and Nuclear Medicine, Royal Prince Alfred Hospital, Missenden Road, Camperdown, NSW, 2050, Australia

**Keywords:** Temporal lobe epilepsy, Neuroinflammation, Rat, Status epilepticus, [^18^F]-PBR111

## Abstract

**Background:**

Recently, inflammatory cascades have been suggested as a target for epilepsy therapy. Positron emission tomography (PET) imaging offers the unique possibility to evaluate brain inflammation longitudinally in a non-invasive translational manner. This study investigated brain inflammation during early epileptogenesis in the post-kainic acid-induced *status epilepticus* (KASE) model with *post*-*mortem* histology and *in vivo* with [^18^F]-PBR111 PET.

**Methods:**

*Status epilepticus* (SE) was induced (*N* = 13) by low-dose injections of KA, while controls (*N* = 9) received saline. Translocator protein (TSPO) expression and microglia activation were assessed with [^125^I]-CLINDE autoradiography and OX-42 immunohistochemistry, respectively, 7 days post-SE. In a subgroup of rats, [^18^F]-PBR111 PET imaging with metabolite-corrected input function was performed before *post*-*mortem* evaluation. [^18^F]-PBR111 volume of distribution (*V*_t_) in volume of interests (VOIs) was quantified by means of kinetic modelling and a VOI/metabolite-corrected plasma activity ratio.

**Results:**

Animals with substantial SE showed huge overexpression of TSPO *in vitro* in relevant brain regions such as the hippocampus and amygdala (*P* < 0.001), while animals with mild symptoms displayed a smaller increase in TSPO in amygdala only (*P* < 0.001). TSPO expression was associated with OX-42 signal but without obvious cell loss. Similar *in vivo* [^18^F]-PBR111 increases in *V*_t_ and the simplified ratio were found in key regions such as the hippocampus (*P* < 0.05) and amygdala (*P* < 0.01).

**Conclusion:**

Both *post*-*mortem* and *in vivo* methods substantiate that the brain regions important in seizure generation display significant brain inflammation during epileptogenesis in the KASE model. This work enables future longitudinal investigation of the role of brain inflammation during epileptogenesis and evaluation of anti-inflammatory treatments.

## Background

Various brain injuries in humans such as neurotrauma, stroke, infection, febrile convulsions and *status epilepticus* (SE) are associated with the acute occurrence of seizures and an increased risk of developing epilepsy. The neurobiological cascades underlying the process of epileptogenesis are still incompletely understood. An important feature regulating the reorganisation of the neuronal network after the occurrence of a neuronal insult is brain inflammation. In recent years, evidence from animal and clinical studies has been accumulating which supports the hypothesis that inflammatory processes within the brain might constitute a common and crucial mechanism in the pathophysiology of seizures and epilepsy [1,2]. This has raised interest in investigating the potential of anti-inflammatory treatment strategies for epilepsy [2,3]. However, to efficiently target brain inflammation, we need to increase our understanding of its ambiguous role in epilepsy, which encompasses both pro-epileptic as well as homeostatic protective mechanisms [1-3].

Epileptogenesis is difficult to study in human patients in a systematic, unbiased prospective manner. Also, the tissue is only available from the very small percentage of temporal lobe epilepsy (TLE) patients that undergo epilepsy surgery. TLE is the most common and severe form of focal epilepsy in adults and is often unsatisfactorily controlled with medical therapy. Animal models offer a distinct advantage to study TLE by allowing the assessment of baseline, developing and chronic epileptic states. Nevertheless, traditional neuroscientific techniques (e.g. histology) can only examine the brain at one time point during the epileptogenic processes in any individual animal. The recent advances in dedicated imaging techniques such as small animal positron emission tomography (PET) allow, for the first time, researchers to study *in vivo* changes during the process of epileptogenesis as well as treatment response by means of serial acquisitions in the same animal [4]. Several groups have recently developed radioligands, which are translocator protein (TSPO) specific and potentially very interesting for clinical use. TSPO or previously named peripheral benzodiazepine receptor (PBR), which is a marker of activated glia, has been detected in surgically resected brain tissue and most recently *in vivo* with PET in patients with TLE [5-8]. Such biomarkers may become of particular value to aide in patient diagnoses and/or stratification, to evaluate the effect of new drug therapies on brain inflammation and for resective surgery of the epileptogenic zone [6,8].

^18^F]-PBR111, with high specificity for the TSPO receptor, has suitable *in vivo* characteristics to be used as a brain inflammation imaging biomarker [9,10]. The aim of this comprehensive study was to investigate brain inflammation during early epileptogenesis in the post-kainic acid-induced SE (KASE) model for TLE with *postmortem* histology and *in vivo*^18^F]-PBR111 small animal PET.

## Methods

### Animals

All experiments were approved by the Institutional Animal Care and Ethics Committee at the Australian Nuclear Science and Technology Organisation (NSW, Australia) and were in strict accordance with the Australian Code of Practice for the Care and Use of Animals for Scientific Purposes.

Adult male Wistar rats (7-week-old) were purchased from the Australian Resources Centre (Canning Vale, Australia) and used for metabolite, *ex vivo* or PET imaging studies. They were acclimatised for 1 week and subsequently single-housed under a 12-h light/dark cycle, in a temperature and humidity controlled environment, with food and water available *ad libitum*. All efforts were made to minimise pain or discomfort to the animals during experimental procedures.

SE was induced in 13 male Wistar rats (286 ± 4 g) as described before [11,12]. Briefly, by step-wise intraperitoneal (i.p.) injections of kainic acid (A.G. Scientific, San Diego, CA, USA) (2.5 to 5 mg/kg, total dose 7.5 to 22.5 mg/kg, mean total dose 12.7 ± 1.4 mg/kg), sustained seizure activity was induced for 4 h, while control rats (280 ± 9 g, *N* = 9) received saline injections (Table 1). Where seizures during SE were too violent and life-threatening, a half dose of diazepam (2 mg/kg) was given during SE. Following 4 h of SE, animals were given diazepam (4 mg/kg, i.p.) to terminate SE. Animals were classified into three groups regarding the severity of the SE employing the scale of Racine [13]: firstly, minor SE was defined as predominantly non-convulsive seizure activity (class I to II seizures) with some forelimb cloni (class III); secondly, medium SE includes all seizure classes up to some occasional class IV seizures (rearing with falling) and continuous automatisms (head nodding, erect tail, hyperactivity, etc.); and thirdly, severe SE includes all seizure classes up to several class V seizures (rearing with falling), tonic clonic convulsions, bouncing and continuous automatisms. Animals were used for metabolite, *ex vivo* and PET imaging studies (Table 1) 7 days post-induction, as previous studies have shown significant brain inflammation/TSPO upregulation in this model during this period [14-16]. 

**Table 1:** Overview of the number of animals and procedures used in each experiment

	**[**^**18**^**F]-PBR111 metabolism*post***-***mortem*evaluation**^**a**^** (*in vitro* [**^**18**^**F]-PBR111**^**b**^**)**	***Ex vivo*[**^**18**^**F]-PBR111**^**c**^	***In vivo*[**^**18**^**F]-PBR111 PET and*post***-***mortem* evaluation**^**d**^
Control (*n*)	4 (1)	1	4
Minor SE (*n*)	3 (0)	0	0
Medium SE (*n*)	3 (1)	1	2
Severe SE (*n*)	0 (0)	1	3

^a^*Post*-*mortem* evaluation was performed on sagittal sections and included *in vitro* [^125^I]-CLINDE autoradiography and OX-42 immunohistochemistry for all animals; ^b^*in vitro* [^18^F]-PBR111 in two representative animals; ^c^*ex vivo* [^18^F]-PBR111 binding was measured in sagittal sections; ^d^*post*-*mortem* evaluation was performed on coronal sections in these animals and included *in vitro* [^125^I]-CLINDE autoradiography, OX-42 immunohistochemistry and Cresyl Echt Violet staining.

### Radiosynthesis of [^125^I]-CLINDE

The radiotracer ^125^I]-CLINDE was synthesised as previously described [17,18] in 65% to 80% radiochemical yield and >97% radiochemical purity. For autoradiography, unlabelled CLINDE was added to ^125^I]-CLINDE to achieve a specific activity of 3.7 GBq/μmol.

### Radiosynthesis of [^18^F]-PBR111

^18^F]-PBR111 was prepared using automated methods on the GE Tracerlab FX_FN_ (GE Healthcare, Milwaukee, WI, USA) as previously published [19]. Briefly, 2 mg of the *p*-toluenesulfonyl precursor of PBR111, dissolved in anhydrous acetonitrile, was added to pre-dried K^18^F]-K_2.2.2_·K_2_CO_3_ complex, prepared from an aqueous ^18^F]-fluoride solution (37 to 150 GBq). The reaction was heated at 100°C for 5 min before the reaction mixture was diluted with mobile phase and purified by preparative reverse phase chromatography. The collected radioactive peak was concentrated using a C^18^-solid phase extraction cartridge with subsequent formulation to a concentration of 20 MBq/100 μL of saline containing less than 1% ethanol for the biological studies. ^18^F]-PBR111 was produced in 25% to 35% radiochemical yield (non-decay corrected) with radiochemical purity exceeding 97% and specific activity of 215 ± 46 GBq/μmol.

### Metabolite studies

To establish a standard curve of [^18^F]-PBR111 metabolism, blood samples were obtained in four control rats (304 ± 10 g) and six KASE rats (312 ± 8 g, minor to medium SE), which were maintained under anaesthesia to mimic the conditions of the PET scan (Table 1). Animals were anaesthetised with 5% isoflurane for induction and 1% to 3% for maintenance, administered in medical oxygen. An incision was made in the inner thigh of the hind leg, and the femoral artery was exposed and loosely tied off after blunt dissection of the surrounding tissue. A polyethylene cannula filled with heparinised saline (20 IU/ml) was placed into the femoral artery and threaded 3 cm proximally.

Animals were injected intravenously with [^18^F]-PBR111 (injected dose (ID) = 14.1 to 38.7 MBq). The tracer was infused via the dorsal penile vein (maximum volume of 0.3 ml) using a Harvard Apparatus Pump 11 plus syringe pump (Holliston, MA, USA) over a constant time interval of 1 min. The line was flushed with saline, and the remaining radioactivity was measured in the line and syringe. Different ranges of tracer mass (0.07 to 4.00 nmol) were injected to evaluate whether this would affect the tracer metabolism. Arterial blood samples (0.2 ml) were withdrawn via a cannula implanted in the femoral artery from 2 to 60 min after the start of injection. The whole blood was then centrifuged at 3,000 rpm for 10 min, and the plasma was collected. Plasma samples (0.1 ml) were weighed and the radioactivity was measured in a Wallac γ-counter. At 2- and 5-min post-injections, the samples were taken in duplicate. One sample was left on the bench for 1 h, and the other was processed immediately to evaluate the stability in whole rat blood. This showed that [^18^F]-PBR111 was not further metabolised *in vitro* in whole rat blood.

Upon termination of blood sampling, the cannula was removed, the artery was tied off and the skin incision was sutured. Animals were given buprenorphine (0.05 mg/kg) as a post-surgical analgesic and killed the following day by carbon dioxide overdose. The brains were immediately removed and snap frozen for *post*-*mortem* evaluation of brain inflammation.

The metabolite analysis was performed using solid phase extraction (SPE) that proved to be a sensitive technique as previously described by [20] to measure the concentration of unchanged ^18^F]-PBR111 in rat plasma [20]. SPE analysis was performed on Oasis cartridges (Waters Oasis® HLB, 30 μm, 60 mg) that were preconditioned with 1 ml ethanol followed by 5 ml water. Rat plasma (100 μl) was mixed with 1 ml of water containing the unlabelled PBR compound (10 nmol in 10 μl CH_3_OH) and KF (200 nmol) and loaded onto the cartridge. Sequentially, the cartridge was washed with 1 ml water, 1 ml acetonitrile/water (20/80, *v*/*v*) and 2 ml acetonitrile. All fractions were collected into separate counting tubes. The radioactivity in each fraction and that remaining on the cartridge was measured with a Wallac γ-counter. Data were expressed as the percentage of unmetabolised parent compound (^18^F]-PBR111) in the plasma sample.

### *Post*-*mortem* evaluation of brain inflammation

Frozen brains from animals studied in the metabolism experiments were used to explore for brain inflammation and TSPO expression. They were cut (sagittal sections 20 μm and 0.9 and 4.3 mm from bregma according to the Paxinos and Watson rat atlas [21]) using a cryostat, thaw-mounted onto the Polysine® microscope slides (LabServ, Australia), and stored at −80°C.

#### Immunohistochemistry

On the slide, sections were post-fixed in 10% buffered formalin (Sigma-Aldrich, St. Louis, MO, USA) for 20 min, washed in 0.1 M phosphate buffered saline (PBS, 6 × 5 min) and the endogenous peroxidases were quenched in 3% hydrogen peroxide solution (in 0.1 M PBS) for 5 min. They were blocked in 3% normal horse serum (in PBS) for 10 min then incubated overnight in mouse anti-CD11b (OX-42, Serotec (Kidlington, Oxford, UK), 1:1,000) in antibody diluent (0.1% bovine serum albumin, 0.2% triton X-100, 2% normal horse serum in 0.1 M PBS). They were washed in PBS (2 × 10 min) then incubated with the secondary antibodies (goat biotinylated anti-mouse IgG, Vector Laboratories (Burlingame, CA, USA), 1:500 in antibody diluent) for 1 h. They were washed in 0.1 M PBS (2 × 15 min) and incubated in 1:1,000 ExtrAvidin peroxidase (Sigma-Aldrich) in antibody diluent. Labelling was visualised using a nickel-intensified diaminobenzidine (DAB) chromogen (0.05% DAB tetrahydrochloride, 0.004% ammonium chloride, 0.2% d-glucose, 0.02% nickel ammonium sulphate, 0.1% glucose oxidase in 0.1 M phosphate buffer), as previously described in [22]. Slides were air-dried, dehydrated through graded ethanol solutions (70%, 95% and 100%), incubated in xylene and coverslipped using Eukit (Proscitech, Queensland, Australia) permanent mounting media. Sections incubated without primary antibodies served as negative controls.

Images were captured using a Q-Imaging Micropublisher 3.3 digital camera (Q-Imaging, Surrey, Canada) on an Olympus BX41 microscope (Olympus America Inc., Melville, NY, USA) with epifluorescence and ImagePro software (Media Cybernetics, Silver Spring, MD, USA). Light microscopy images were captured using ×2 and ×10 objectives. Image montages were created from images captured using a ×2 objective and were non-destructively positioned using Photoshop CS4 extended (Adobe, USA). Qualitative analysis of OX-42-labelled brain sections involved blinded visual inspection for OX-42 increased signal in the different brain regions in KASE compared to control groups.

#### In vitro [^125^I]-CLINDE and [^18^F]-PBR111 binding

TSPO binding density was assessed using the selective TSPO ligands, ^125^I]-CLINDE and ^18^ F]-PBR111 as described previously [9,23]. Briefly, adjacent sets of unfixed sagittal sections were incubated in triplicate with 3 nM ^125^I]-CLINDE or 4 nM ^18^F]-PBR111 in 50 mM Tris buffer (pH 7.4 at 4°C) for 1 h. Non-specific binding was assessed with co-incubation with 10 μM PK11195 (Sigma-Aldrich). Sections were washed in ice cold Tris buffer (2 × 2 min), then distilled water (1 min), before being air dried on a slide warmer at 37°C. They were exposed to film, with ^14^C] polymer standards (ARC, USA) for calibration of optical density to becquerel per milligram activity. Films were scanned using a BioRad GS-800 calibrated densitometer (Bio-Rad Laboratories, Hercules, CA, USA), and exposure times were chosen such that the optical density range of the sections was within the pseudolinear response range of the film.

Autoradiographic images were calibrated (cubic fit between optical density and [^14^C]-standard values) and quantified using ImageJ ( http://rsbweb.nih.gov/ij/). After visual inspection of the autoradiographic films, regions with high TSPO binding namely the hippocampus, thalamus and amygdala were chosen for quantification, as well as a region with limited binding namely the inferior colliculus. Regions of interest were drawn blinded for control and KASE groups, converted to kilobecquerel per milligram and specific binding calculated.

#### Ex vivo binding

To evaluate [^18^F]-PBR111 for *in vivo* TSPO binding, the following *ex vivo* autoradiography study was performed (Table 1). One control animal (275 g) and two kainic acid-treated animals (medium and severe SE, 263 to 296 g) were injected i.v. with 37 MBq [^18^F]-PBR111 under isoflurane anaesthesia (5% induction, 1% to 3% maintenance in medical oxygen). Thirty minutes later, the animals were killed with carbon dioxide; the brains were extracted and snap frozen. The brain sections were exposed on phosphor imaging plates (SR 2025, FujiFilm, Japan) overnight. Phosphor imaging plates were scanned using a BAS-5000 Bioimaging Analyser (Fujifilm, Japan) to digitise the autoradiograms for presentation.

### [^18^F]-PBR111 PET imaging of subsequent brain inflammation and *post*-*mortem* validation

Four control (317 ± 8 g) and five KASE (290 ± 8 g) animals were imaged *in vivo* with [^18^F]-PBR111 PET (Table 1). The KASE group displayed medium to severe SE during induction.

#### [^18^F]-PBR111 PET imaging

Seven days post-SE, rats were anaesthetised for PET imaging using isoflurane (induction 5%, maintenance 1% to 3%, in medical oxygen), and body temperature was maintained using a feedback-regulated heating pad. A catheter was implanted in the femoral artery as described above. The animal was then placed into the field-of-view of the INVEON PET/CT scanner (Siemens Medical Solutions, Knoxville, TN, USA) [24], and physiological parameters (heart rate, respiration and body temperature) were monitored (BioVet, m2m Imaging Corp., Cleveland, OH, USA) for the entire scanning period. To avoid movement and fix tubing, the animal was restrained in the supine position using a webbed sock and adhesive tape.

The PET scan was started 20 to 30 s before the 1-min infusion of radiotracer (ID = 12.5 to 29.0 MBq, 0.05 to 0.29 nmol, max 0.4 ml in volume, as described above) for the duration of 60 min. Femoral arterial blood samples (approximately 50 μl/sample) were taken at 15-s intervals for 4 min after the commencement of the PET scan, and then at 10-, 20-, 30- and 50-min post-injections. In the last sample (100 μl), metabolites were analysed to confirm the standard metabolite correction and to calculate the partition coefficient at 50 min. Heparinised saline (20 IU) was administered after blood sampling to maintain the blood volume and to prevent hypotension. Plasma samples were collected after 10-min centrifugation of whole blood at 3,000 rpm. Whole blood and plasma samples were counted in a Wallac γ-counter, decay-corrected and weight-normalised. At the end of the PET scanning, a 10-min 70 kV/500 μA computed tomography (CT) scan was performed on each animal (for co-registration with PET data and magnetic resonance imaging (MRI) atlas).

In a subset of animals (one control and three KA-treated animals), Evans Blue (EB, 2%, 4 ml/kg) was injected through the femoral artery catheter under isoflurane anaesthesia to assess the integrity of the blood brain barrier macroscopically [25,26]. Animals were then sacrificed at the end of the scan using carbon dioxide overdose. The brains were immediately removed and frozen in liquid nitrogen and stored at −80°C.

#### PET image analysis

Coincidence events were acquired into list mode files and then histogrammed into 31 consecutive frames of variable duration (8 × 15 s, 6 × 20 s, 6 × 60 s, 3 × 180 s, and 5 × 500 s). The volumes were then reconstructed with an iterative algorithm 3D-OSEM/FastMap. PET images (128 × 128 × 159) were co-registered with CT scans and mapped to a MRI-based rat brain atlas [27]. Left and right time activity curves (TACs) were derived from eight VOIs such as the frontal cortex, striatum, parietal cortex, hippocampus, thalamus, amygdala, occipital cortex and colliculi using the Anatomist package ( http://brainvisa.info). Also, the hypothalamus, brainstem, pons and cerebellum were extracted from the reconstructed images. Data were then converted into activity concentration (kBq/ml) using cross calibration factors.

Firstly, a two-compartment model was used to assess the ‘apparent’ volume of distribution (*V*_t_), or the ‘volume’ of the tracer would occupy if the tracers were to adopt the same concentration in the tissue as in the blood. For each VOI, *V*_t_ was calculated using their TACs, the metabolite-corrected plasma curve as input function and the whole blood curve to model the kinetics of the vascular fraction (5%) in the cerebral tissue.

Secondly, the ratio between the VOI activity and the metabolite-corrected plasma activity (r_50′_) was calculated to determine its appropriateness for longitudinal studies without the need of a plasma input function. At a late time point, r_50′_ was calculated as the ratio of the activity in the hippocampus, thalamus and amygdala (frame 49 to 54 min) over the metabolite-corrected plasma activity at 50 min. Metabolite correction derived from the standard curve was indicated as r_50′(SC)_, while metabolite correction derived from the blood sample was indicated as r_50′_.

#### Post-mortem verification of altered TSPO density, microglial activation and cell loss

Coronal brain sections (20 μm, −3.24 mm from bregma according to [21]) were cut using a cryostat, thaw-mounted onto Polysine® microscope slides (LabServ, Australia), and stored at −80°C. During cutting, pictures were taken in EB-injected animals to evaluate extravasation macroscopically. Adjacent sets of unfixed rat brain sections were analysed in triplicate for quantitative autoradiography for TSPO with ^125^I]-CLINDE, OX-42 (microglial activation) immunohistochemistry and cresyl violet (cell loss).

Methodology regarding immunohistochemistry was performed as above. Semi-quantitative analysis included visual scoring of OX-42 signal as follows: mild (+), moderate (++) and intense (+++) OX-42 signals. For autoradiography, a preincubation step of 15 min in Tris buffer (50 mM, pH 7.4) was added to remove exogenous TSPO ligand. TSPO binding was assessed as described above in the cortex, corpus callosum, hippocampus and hippocampal subregions (CA1, CA2 and CA3/hilus), thalamus, hypothalamus, amygdala and piriform cortex. Histological verification of cell loss in hippocampal pyramidal cell layers (CA1, CA2, CA3 and hilus) was performed on Nissl-stained sections (using Cresyl Echt Violet) [21] blinded for the treatment group.

### Statistical analysis

Statistical analysis was performed with Prism v5.0d for Mac (GraphPad Software Inc., La Jolla, CA, USA). Biological half-life of the radiotracer was calculated using non-linear fitting with a two-phase decay, and comparison of fits was performed. TSPO binding between the control, minor SE and medium SE groups was compared using one-way ANOVA with Tukey *post hoc* tests for the different brain regions. For the PET studies, *V*_t_ and *post*-*mortem* TSPO binding between the control and KASE groups were compared using a Student's *t* test with Welch correction if variance was unequal. A value of *P* < 0.05 was considered significant; a trend was indicated with *P* < 0.1. Correlations between *V*_t_, r_50′_ and autoradiography were obtained with Pearson *r* test. The percentage of difference in the KA group was calculated as (KA − control) / control × 100. Data are expressed as mean ± standard error of the mean (SEM).

## Results

### Standard metabolite correction

**Figure 1:**
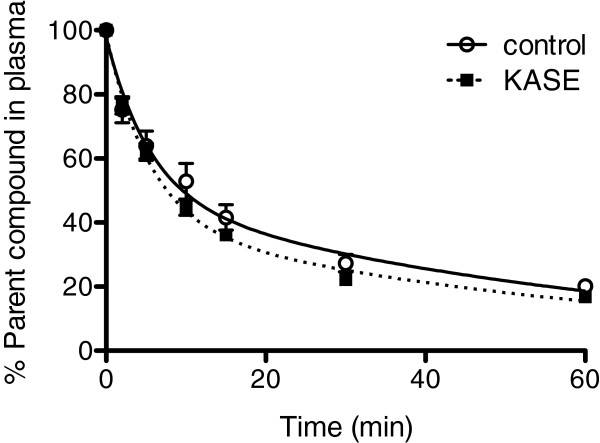
**Metabolism of[**^**18**^**F]-PBR111.** In the plasma of control (*N* = 4) and KASE (*N* = 6) rats, metabolism of [^18^F]-PBR111 was expressed as the percentage of parent compound.

Metabolism of the parent ^18^F]-PBR111 compound varied little among the different animals and was not depending on the injected tracer mass. Although there was a small difference between the control and KASE animals in the percentage of parent compound over time in the plasma, these radioligand metabolism curves were not significantly different (Figure 1). Data were fitted with a two-exponential fit (*R*^2^ > 0.95), and the biological short and long half-lifes were in agreement with Katsifis et al. [20]. 

### *In vitro* and *ex vivo* TSPO binding

**Figure 2:**
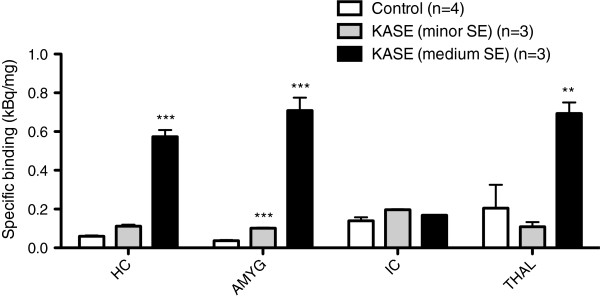
**TSPO binding 7 days post**-**KASE was depending on the severity of SE.***In vitro * TSPO binding was performed on sagittal sections and is expressed as specific binding of [^125^I]-CLINDE (kBq/mg). HC, hippocampus; AMYG, amygdala; IC, inferior colliculus; THAL, thalamus. Data are expressed as mean ± SEM; *P* < 0.01 (double asterisk) and *P* < 0.001 (triple asterisk).

After injection of KA, animals displayed different levels of severity of SE during the 4-h induction period. The severity of the SE correlated with the *in vitro* TSPO radioligand [^125^I]-CLINDE binding. Animals that experienced medium severity of SE showed significant increases in TSPO binding in the hippocampus (*P* < 0.01), amygdala (*P* < 0.001) and thalamus (*P* < 0.01) (Figure 2). The minor SE group displayed significantly increased TSPO binding in the amygdala only (*P* < 0.001) and to a much lower degree than the medium SE group (Figure 2). The inferior colliculus, a region not directly involved in seizure generation was not affected (Figure 2).

**Figure 3:**
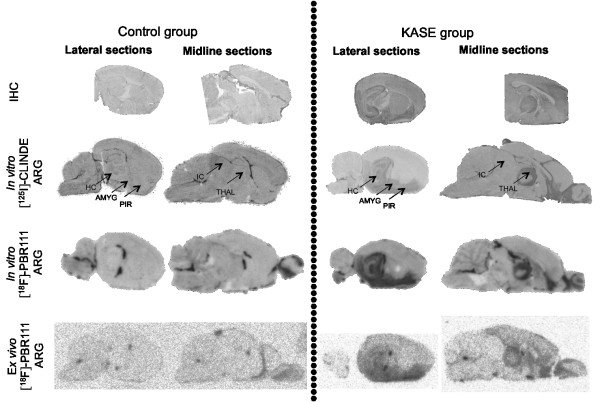
**Brain inflammation assessed by immunohistochemistry.***In vitro* and *ex vivo* TSPO autoradiography on sagittal sections in representative control and KASE (medium SE) animals demonstrating higher signal in the hippocampus, piriform cortex, amygdala and thalamic nuclei post-KASE. IHC, immunohistochemistry; ARG, autoradiography, HC, hippocampus; AMYG, amygdala; PIR, piriform cortex; IC, inferior colliculus; THAL, thalamus.

*In vitro* [^125^I]-CLINDE binding corresponded closely with OX-42 immunohistochemistry: brain regions including the hippocampus, amygdala, thalamus and piriform cortex were labelled with both methods in KASE animals (Figure 3). In both control and KASE animals, ependymal cells of the ventricles and the olfactory bulb showed TSPO expression (Figure 3). The same brain regions were labelled after *in vitro* and *ex vivo* [^18^F]-PBR111 autoradiography compared with *in vitro* [^125^I]-CLINDE autoradiography (Figure 3).

### [^18^F]-PBR111 PET imaging with *post*-*mortem* validation

**Figure 4:**
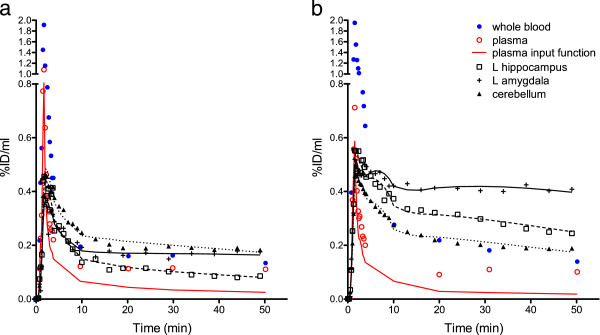
**Time activity curves** (**TACs**) **of[**^**18**^**F]-PBR111.** TACs were expressed as the percentage injected dose per milliliter (%ID/ml) of plasma and several brain regions in the representative control (**a**) and KASE (severe SE) (**b**) rats. The increase in the amplitude of the cerebellum is proportional to the plasma in both animals, while the increase in amygdala is much higher in the KASE compared to the control rat. Note that the kinetics of the radioligand is also slower in the amygdala of the KASE animal.

KASE animals, used for the PET imaging study, displayed medium to severe SE during induction. Input functions were corrected using the above-derived standard metabolite correction curves fitted with two-exponential fit. As in the previous experiment, only the control, minor and medium SE animals were investigated; we also compared the metabolism between the control and severe SE animals. The percentage of parent compound at 50-min post-injection was not significantly different between the control (20.8 ± 1.3%) and severe SE (20.1 ± 1.3%) animals. Representative TACs for plasma and a subset of brain regions are presented in Figure 4, and mean *V*_t_ values for all regions are listed in Table 1. *V*_t_ did not differ significantly between the left and right hemispheres in the control and KASE-treated rats. In one KASE animal, *V*_t_ was not elevated, and this animal was omitted from the comparison between the control and KASE groups. Significant bilateral brain increases of *V*_t_ were found in the left and right striatum (180% and 156%, respectively, *P* < 0.05), right parietal cortex (136%, *P* < 0.05), left and right hippocampus (154%, *P* < 0.01 and 159%, *P* < 0.05, respectively), right thalamus (155%, *P* < 0.05), left and right amygdala (288%, *P* < 0.01 and 284%, *P* < 0.05, respectively) and right occipital cortex (62%, *P* < 0.05) (Table 1). A trend for a significant difference (*P* < 0.1) was found in the left and right frontal cortices (108% and 98%, respectively), left parietal cortex (110%), left thalamus (173%) and hypothalamus (104%). In the following regions, no significant changes were observed after KA treatment: the left occipital cortex, colliculi, brainstem, pons and cerebellum.

**Figure 5:**
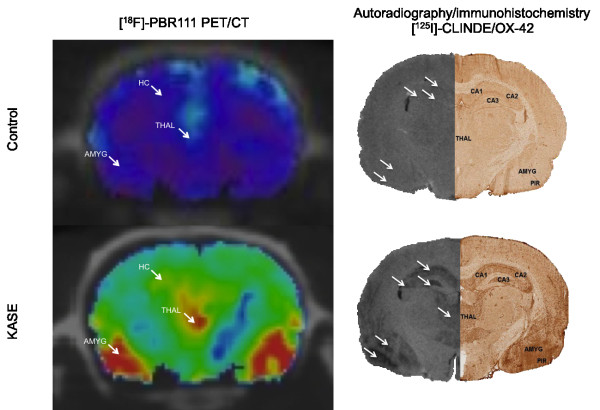
**Evaluation of brain inflammation by the use of*in vivo*[**^**18**^**F]-PBR111 PET imaging** (**coronal plane).** Corresponding *post*-*mortem* autoradiography and immunohistochemistry on the coronal sections in the representative control and KASE (severe SE) rats, 7 days post-SE induction are shown. Arrows indicate the different brain regions named on the ipsilateral hemisphere. HC, hippocampus; THAL, thalamus; AMYG, amygdala; PIR, piriform cortex.

**Figure 6:**
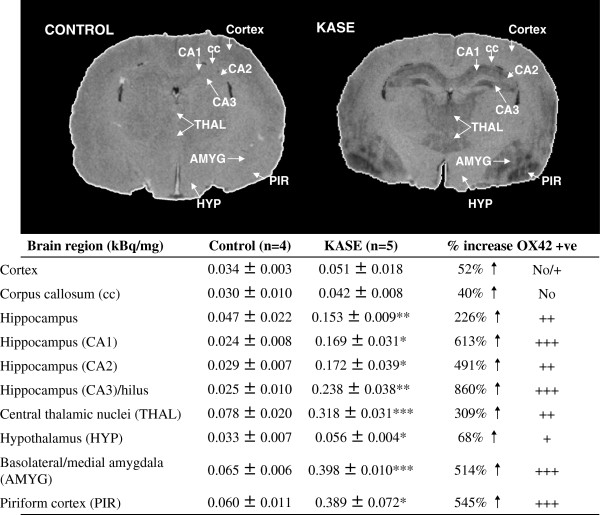
**[**^**125**^**I]-CLINDE autoradiography images and quantitative analyses.** [^125^I]-CLINDE autoradiography images of the representative control and KASE (severe SE) rats and quantitative analysis of [^125^I]-CLINDE specific binding (kBq/mg) in the control (*N* = 4) and KASE (*N* = 5) groups of dorsal hippocampal sections are shown. Data were pooled for medium (*N* = 2) and severe SE (*N* = 3) groups as qualitative inspection indicated that there was no difference in TSPO binding between the medium and severe groups. OX-42 + ve, OX-42 positive staining. Upward arrow indicates increase; plus sign, mild; double plus sign, moderate; and triple plus sign, intense OX-42 signals. Data are expressed as mean ± SEM; *P* < 0.05 (asterisk), *P* < 0.01 (double asterisk) and *P* < 0.001 (triple asterisk).

Increased [^18^F]-PBR111 binding was measured *in vivo* with PET which corresponds with the brain regions with increased brain inflammation identified with [^125^I]-CLINDE autoradiography and OX-42 immunohistochemistry (Figure 5). Increased [^125^I]-CLINDE binding was also observed in the KASE animal without increased [^18^F]-PBR111 *V*_t_. In the whole hippocampus, a significant increase of 226% in [^125^I]-CLINDE binding was found (Figure 6). When the subregions of the hippocampus were analysed on the high-resolution autoradiographic images, binding differences between the control and KASE animals in CA1 (613%), CA2 (491%) and CA3 (860%) were higher compared to the dorsal hippocampus as a whole (226%) (Figure 6). Other brain regions analysed, i.e. the cortex, central thalamic nuclei, hypothalamus, basolateral/medial amygdala and piriform cortex showed significant increases in [^125^I]-CLINDE binding compared to controls in agreement with the OX-42 positive signal (Figure 6) and *in vivo* PET (Table 2). In corpus callosum, TSPO binding was not significantly different from the control animals. 

**Table 2:** Tissue distribution volumes of different VOIs

**Brain region** (***V***_**t**_)	**Hemisphere**	**Control** (***N*** = **4**)	**KASE** (***N*** = **4**)	**Increase** (%)
Frontal cortex	*Left*	5.4 ± 1.0	11.3 ± 2.4^§^	108
	*Right*	5.7 ± 1.2	11.2 ± 2.4^§^	98
Striatum	*Left*	2.8 ± 0.6	8.0 ± 1.2*	180
	*Right*	3.0 ± 0.5	7.8 ± 1.1*	156
Parietal cortex	*Left*	4.5 ± 0.6	9.5 ± 1.1^§^	110
	*Right*	4.9 ± 0.8	11.6 ± 2.0*	136
Hippocampus	*Left*	3.6 ± 0.5	9.1 ± 1.1**	154
	*Right*	3.6 ± 0.4	9.4 ± 1.6*	159
Thalamus	*Left*	2.6 ± 0.5	7.0 ± 1.8^§^	173
	*Right*	2.8 ± 0.5	7.1 ± 1.4*	155
Hypothalamus		4.0 ± 0.7	8.2 ± 1.4^§^	104
Amygdala	*Left*	5.3 ± 0.8	20.6 ± 2.8**	288
	*Right*	6.1 ± 0.7	23.4 ± 4.8*	284
Occipital cortex	*Left*	9.8 ± 3.4	15.1 ± 3.5	53
	*Right*	9.3 ± 1.4	15.0 ± 1.4*	62
Colliculi	*Left*	4.0 ± 1.0	5.8 ± 1.1	44
	*Right*	4.1 ± 1.1	6.9 ± 1.8	70
Brainstem		4.7 ± 0.8	6.7 ± 1.0	41
Pons		4.2 ± 0.9	7.5 ± 1.8	78
Cerebellum		7.5 ± 0.8	10.6 ± 1.3	43

Data are expressed as mean ± SEM; ^§^*P* < 0.1, *P < 0.05 and **P < 0.01 compared to control.

**Figure 7:**
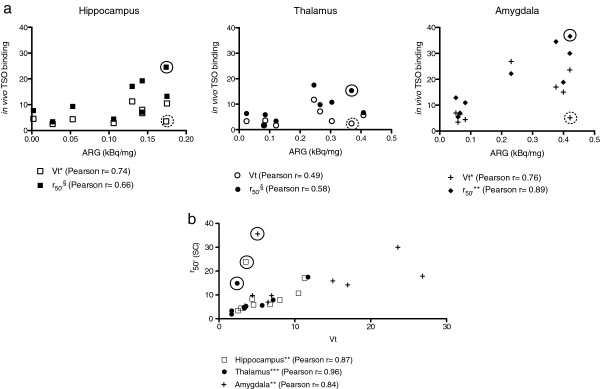
***In vivo * and *in vitro * imaging correlations.** (**a**) Correlation between * in vivo * ([^18^F]-PBR111 *V*_t_ and r_50′_) and *in vitro* ([^125^I]-CLINDE autoradiography) measurements of TSPO binding for the hippocampus, thalamus and amygdala. (**b**) Correlation between [^18^F]-PBR111 * V *_t_ and r_50′(SC)_*in vivo* measurements of TSPO binding for the hippocampus, thalamus and amygdala. Circles indicate the *V*_t_ [^18^F]-PBR111-negative animal, with, in (a), open circles showing the *V*_t_ values for this animals and closed circles showing the r_50′(SC)_ value of this animal. This animal was excluded for statistical correlation between *V*_t_ and ARG, and between *V*_t_ and r_50′(SC)_. ARG, autoradiography. Asterisk indicates *P* < 0.05; double asterisk, *P* < 0.01; triple asterisk, *P* < 0.001; and section sign, *P* < 0.1.

A correlation between *V*_t_ and r_50′_ and autoradiography binding was assessed for the hippocampus, thalamus and amygdala. There was a discrepancy in the results for one KASE rat, which did not show increased *V*_t_, as mentioned above, but nevertheless had elevated r_50′_ and [^125^I]-CLINDE autoradiography binding (Figure 7). The strongest correlation between *in vivo* measurements *V*_t_ (excluding the [^18^F]-PBR111-negative animal, Pearson *r* = 0.76, *P* < 0.05) and r_50′_ (Pearson *r* = 0.89, *P* < 0.01) with autoradiography was found for the amygdala (Figure 7). Highly significant correlations were found between r_50′(SC)_ and *V*_t_ for the three brain regions analysed (hippocampus: Pearson *r* = 0.87, *P* < 0.01; thalamus: Pearson *r* = 0.96, *P* < 0.001 and amygdala: Pearson *r* = 0.84, *P* < 0.01) after exclusion of the [^18^F]-PBR111-negative animal (Figure 7).

Obvious cell loss was not present in any of the animals in CA1, CA1 and CA3 regions, except for one KASE animal, which displayed cell loss in CA4. Macroscopic EB extravasation into the brain was not observed in the subset of animals inspected.

## Discussion

Brain inflammation was demonstrated and quantified utilising both terminal histological/autoradiographic techniques as well as *in vivo* small animal PET imaging 7 days post-KA-induced SE. This study emphasised that [^18^F]-PBR111, a specific TSPO binding radioligand, is a promising radiotracer for imaging brain inflammation during epileptogenesis. Particularly, TSPO overexpression was observed after the induction of *status epilepticus* in several brain regions with a well-established role in seizure generation and/or propagation. Interestingly, the severity of the *status epilepticus* corresponded with the extent of TSPO overexpression.

Recent animal studies have demonstrated that acutely evoked seizures rapidly induce inflammatory mediators in seizure prone brain regions (reviewed by [1]). Our results are in line with the previous histological studies which have demonstrated that the KASE model constitutes an excellent model to study the involvement of brain inflammation/translocator protein expression in the genesis of epilepsy [14-16,28]. Because spatial information is revealed by the techniques used in our study (autoradiography of the brain sections and small animal PET imaging), we were able to investigate the regional pattern of increased TSPO expression. We have found that the severity of the SE correlated well with the extent of the brain inflammation, while a dose–response relationship between KA dose and TSPO expression was not observed. These data suggest a direct relationship between seizure activity and subsequent brain inflammation 1 week post-SE. More specifically, in animals that displayed minor SE, significant TSPO overexpression was not only considerably lower compared to the medium SE group but also limited to the amygdala. Several brain regions with high seizure susceptibility, such as the hippocampus, amygdala and piriform cortex showed significantly increased TSPO levels in animals that underwent medium to severe SE, which is in line with the previous reports [16,28]. Due to SE timing in the animals and the differences in tissue sectioning and analysis, it was not always possible to present the complete imaging data for each SE state. Because in the first group, brain sections were cut sagitally to have an overview of the complete brain, we believe this data cannot be directly compared against the first cohort of animals, which was cut coronally to focus on the hippocampus, thalamus and amygdala. Qualitatively, based on the results of the PET study with two medium and three severe animals, the data indicate that there was no difference between TSPO binding/inflammation between medium and severe groups. Physiologically, this may be interpreted as follows: once a certain degree of brain excitation/excitotoxicity is reached, TSPO expression does not further increase. However, more studies would be needed to confirm this.

The results from the first *in vitro* experiment were confirmed with *in vivo* [^18^F]-PBR111 PET imaging. Interestingly, in addition to key regions for seizure generation in temporal lobe epilepsy, *in vivo* PET identified several other brain regions with moderate levels of increased TSPO binding such as the hypothalamus (Table 1) which was in case of the hypothalamus confirmed by *in vitro* binding and immunohistochemistry in the same animals (Table 2).

Only a limited number of animals (*N* = 4 to 5 per group) were evaluated due to the complexity of this study as small animal PET imaging with arterial input function in this disease model requires the extensive coordination of a great number of multi-disciplinary procedures namely the induction of the model, tracer synthesis, animal preparation/anaesthesia, PET scanning, blood sample handling, metabolite analysis, blood brain barrier integrity test, animal scarification and histology. The disadvantage of using a relatively small sampling size is that changes in other brain regions, e.g. the pons, that are less pronounced could be missed due to type II errors.

Altar and Baudry [28] hypothesised that increased TSPO expression in the post-KASE model was a result of glial hypertrophy and proliferation in response to cellular necrosis as TSPO increases were observed over a period of 1 to 2 weeks in regions prone to cell loss. Our results indicate that TSPO expression could be a more sensitive and early biomarker for epileptogenesis than cell loss as substantial TSPO increases were not accompanied by vast cell loss 1 week post-KASE. Indeed, Vezzani and Friedman [29] suggested that inflammation might be intrinsic to the epileptogenic process and therefore potentially an epileptogenesis biomarker.

Several studies utilising resected tissue from chronic epileptic patients have shown increased TSPO binding [5-7]. More recently, studies employing ^11^C]-PK11195, although having inferior PET imaging characteristics (signal-to-noise ratio) than ^18^F]-PBR111 [10], succeeded in identifying focal and diffuse regions of activated microglia in Rasmussen's encephalitis [30] and areas of focal cortical dysplasia [31]. In temporal lobe epilepsy patients, ^11^C]-PBR28, a recently developed carbon-11-labelled TSPO ligand, showed increased binding ipsilateral to the seizure focus [8]. This recent evidence and our preclinical study implicate that TSPO imaging could be a promising diagnostic biomarker: firstly, by helping to identify the degree of inflammation and the affected regions in patients and, therefore, TSPO PET imaging could identify individuals that would benefit from anti-inflammatory treatment; secondly, TSPO PET imaging could act as a biomarker and possibly a surrogate marker for preclinical and clinical studies investigating how drugs and potentially anti-inflammatory drugs affect brain inflammation/disease condition. Nevertheless, a better understanding of the inflammatory processes will be needed before these applications become clinical utilities. Preclinical TSPO small animal PET imaging may play an important role in such validation studies and increase our knowledge about the development of brain inflammation during epileptogenesis and its consequences.

However, there are several factors that may impede preclinical and routine clinical TSPO imaging. Firstly, it has been demonstrated that in some individuals, TSPO binding by several TSPO specific radioligands is negligible [8,32]. This has been attributed to a low binding site, and three subpopulations were identified: high affinity binders (HAB), mixed affinity binders (MAB) and low affinity binders (LAB) [32]. Indeed, Owen et al. showed that TSPO Ala147Thr polymorphism predicts PBR28 binding affinity in human platelets [33]. Therefore, TSPO brain images might not be conclusive for all individual epilepsy patients. Even though ^18^F]-PBR111 affinity differences between MAB and LAB are less pronounced than for TSPO ligands such as ^11^C]-PBR28 and ^11^C]-PBR06 [32], also in our study there was one KASE rat with conflicting data. More specifically, while *postmortem* microglia pathology was observed, ^18^F]-PBR111 *V*_t_ was not increased. This may relate to a higher dissociation constant (*k*_off_) in accordance with the previously formulated existence of HAB and LAB populations [32]. *In vitro*^125^I]-CLINDE autoradiography may be less susceptible to changes in *k*_off_ as autoradiography is generally performed at saturating levels of radioligand concentration. Nevertheless, this does not explain the discrepancy with the *in vivo*^18^F]-PBR111 r_50′_ data. Another explanation could be a difference in tracer influx/efflux (k_1_/k_2_), a technical issue with the input curve or a combination of both which is masking the expected increase in *V*_t_ in this KASE animal. Possibly, a late measurement is less susceptible for these alterations than a kinetically modelled *V*_t_, resulting in a divergence of both *in vivo* measurements. Overall, r_50′_ correlated well with *V*_t_ and ^125^I]-CLINDE autoradiography. This measurement may provide a valuable non-invasive tool for longitudinal studies.

Previous studies showed a dynamic and transient disruption of the blood brain barrier (BBB) during epileptogenesis and the chronic epileptic state [25,34], which may cause tracer perfusion differences in the KASE model and therefore differences in tracer influx (k_1_), as well as tracer efflux (k_2_). Although, *postmortem* verification of EB extravasation in a subset of animals did not reveal macroscopic disruption of the BBB at the time of scanning in our study, this might be a potential confounder. This question merits further investigation for instance by using more complex experiments, such as several injections of different masses of tracer to identify all the parameters of the compartmental model including the apparent affinity (KdVr) and density (Bmax) [35,36]. We are planning such follow-up studies for ^18^F]-PBR111 in the control and KASE animals. However, these types of protocols are invasive and cumbersome and, therefore, not appropriate for longitudinal follow-up.

Further, intrinsic factors to epilepsy such as partial volume effects due to hippocampal sclerosis may result in underestimation of TSPO expression *in vivo*. As the inflammatory region may not exactly match the anatomical structure, a pixel-wise partial volume correction strategy merits consideration [37]. Indeed, autoradiographic images clearly demonstrate that TSPO expression is not homogenous in the hippocampus with its layered architecture. This may explain the lower correlation of the *in vivo* measurements of the hippocampus with autoradiography compared to the amygdala. Also in the thalamus, TSPO expression was confined to specific thalamic nuclei embracing the central part of the thalamus only. With VOI-based analysis, it is not possible to extract this region from the MRI atlas, which may contribute to the lower correlation between *in vivo* and *in vitro* measurements of the thalamus. Statistical parametric mapping (SPM) with a voxel-wise comparison between the control and KASE groups based on a simplified quantification output such as the r_50′_ might further improve the data. SPM on the other hand would need to be conducted in sufficiently powered studies to reach significant differences due to the need for correction of multiple comparisons.

## Conclusion

We have been able to demonstrate significant increases in TSPO binding in brain regions relevant for epilepsy employing both *post*-*mortem* autoradiography and *in vivo* [^18^F]-PBR111 small animal PET imaging while providing a non-invasive quantification method. This study sets a much-needed translational framework for studying brain inflammation and potentially for evaluating novel anti-inflammatory therapies using a PET imaging approach intended to facilitate the transfer of knowledge from bench side to clinical application.

## Abbreviations

CT: computed tomography; EB: Evans blue; ID: injected dose; i.p.: intraperitoneal; KASE: kainic acid-induced *status epilepticus*; MRI: magnetic resonance imaging; PBR: peripheral benzodiazepine receptor; PBS: phosphate buffered saline; PET: positron emission tomography; SE: *status epilepticus*; SPE: solid phase extraction; TAC: time activity curve; TLE: temporal lobe epilepsy; TSPO: translocator protein; *V*_t_: tissue volume of distribution; VOI: volume of interest; r_50′_: VOI/metabolite-corrected plasma activity ratio at 50 min post-tracer injection; r_50′(SC)_: VOI/metabolite-corrected plasma activity ratio at 50 min post-tracer injection with plasma values derived from the standard curve.

## Competing interests

The authors declare that they have no competing interests.

## Authors' contributions

For this manuscript, the 11 authors contributed profoundly. SD, PDC, PB, FM, CL, AK and MCG conceived and designed the study. TP and AK validated and synthesised the tracer; SD, PDC, GLR, PB, MQ, FM, AK and MCG acquired the data. SD, PDC, TP, GLR, HA, PB, MQ and MCG quantified the data. SD, PDC, HA, FM, CL, AK and MCG analysed and interpreted the data; SD and PDC wrote the manuscript; SD, PDC, TP, GLR, PB, MQ, FM, CL, AK and MCG critically edited and revised the manuscript. All authors read and approved the final manuscript.

## References

[B1] Vezzani A French J Bartfai T Baram TZ The role of inflammation in epilepsy Nat Rev Neurol 2011 7 31 40 10.1038/nrneurol.2010.178 21135885 PMC3378051

[B2] Dedeurwaerdere S Friedmand A Fabene PF Mazarati A Murashima YL Vezzani A Baram TZ Finding a better drug for epilepsy: anti-inflammatory targets Epilepsia 2012 53 5 1113 1118 22691043 10.1111/j.1528-1167.2012.03520.x PMC3389561

[B3] Ravizza T Balosso S Vezzani A Inflammation and prevention of epileptogenesis Neurosci Lett 2011 497 3 223 230 10.1016/j.neulet.2011.02.040 21362451

[B4] Dedeurwaerdere S Jupp B O'Brien TJ Positron emission tomography in basic epilepsy research: a view of the epileptic brain Epilepsia 2007 48 Suppl 4 56 64 17767576 10.1111/j.1528-1167.2007.01242.x

[B5] Sauvageau A Desjardins P Lozeva V Rose C Hazell AS Bouthillier A Butterwort RF Increased expression of "peripheral-type" benzodiazepine receptors in human temporal lobe epilepsy: implications for PET imaging of hippocampal sclerosis Metab Brain Dis 2002 17 3 11 10.1023/A:1014044128845 11893007

[B6] Johnson EW de Lanerolle NC Kim JH Sundaresan S Spencer DD Mattson RH Zoghbi SS Baldwin RM Hoffer PB Seibyl JP Innis RB "Central" and "peripheral" benzodiazepine receptors: opposite changes in human epileptogenic tissue Neurology 1992 42 811 815 10.1212/WNL.42.4.811 1314342

[B7] Kumlien E Hilton-Brown P Spannare B Gillberg PG In vitro quantitative autoradiography of [3 H]-L-deprenyl and [3 H]-PK 11195 binding sites in human epileptic hippocampus Epilepsia 1992 33 610 617 10.1111/j.1528-1157.1992.tb02336.x 1321029

[B8] Hirvonen J Kreisl WC Fujita M Dustin I Khan O Appel S Zhang Y Morse C Pike VW Innis RB Theodore W Increased in vivo expression of an inflammatory marker in temporal lobe epilepsy J Nucl Med 2012 53 2 234 240 10.2967/jnumed.111.091694 22238156 PMC3832892

[B9] Fookes CJ Pham TQ Mattner F Greguric I Loc'h C Liu X Berghofer P Shepherd R Gregoire MC Katsifis A Synthesis and biological evaluation of substituted [18 F]imidazo[1,2-a]pyridines and [18 F]pyrazolo[1,5-a]pyrimidines for the study of the peripheral benzodiazepine receptor using positron emission tomography J Med Chem 2008 51 3700 3712 10.1021/jm7014556 18557607

[B10] Van Camp N Boisgard R Kuhnast B Theze B Viel T Gregoire MC Chauveau F Boutin H Katsifis A Dolle F Tavitian B In vivo imaging of neuroinflammation: a comparative study between [(18)F]PBR111, [ (11)C]CLINME and [ (11)C]PK11195 in an acute rodent model Eur J Nucl Med Mol Imaging 2010 37 962 972 10.1007/s00259-009-1353-0 20069292

[B11] Vivash L Tostevin A Liu DS Dalic L Dedeurwaerdere S Hicks RJ Williams DA Myers DE O'Brien TJ Changes in hippocampal GABAA/cBZR density during limbic epileptogenesis: relationship to cell loss and mossy fibre sprouting Neurobiol Dis 2011 41 227 236 10.1016/j.nbd.2010.08.021 20816783

[B12] Dedeurwaerdere S van Raay L Morris MJ Reed RC Hogan RE O'Brien TJ Fluctuating and constant valproate administration gives equivalent seizure control in rats with genetic and acquired epilepsy Seizure 2011 20 72 79 10.1016/j.seizure.2010.10.011 21093310

[B13] Racine RJ Modification of seizure activity by electrical stimulation II. Motor seizure Electroencephalogr Clin Neurophysiol 1972 32 281 294 10.1016/0013-4694(72)90177-0 4110397

[B14] Veenman L Leschiner S Spanier I Weisinger G Weizman A Gavish M PK 11195 attenuates kainic acid-induced seizures and alterations in peripheral-type benzodiazepine receptor (PBR) protein components in the rat brain J Neurochem 2002 80 917 927 10.1046/j.0022-3042.2002.00769.x 11948256

[B15] Veiga S Azcoitia I Garcia-Segura LM Ro5-4864, a peripheral benzodiazepine receptor ligand, reduces reactive gliosis and protects hippocampal hilar neurons from kainic acid excitotoxicity J Neurosci Res 2005 80 129 137 10.1002/jnr.20430 15696538

[B16] Najm I El-Skaf G Massicotte G Vanderklish P Lynch G Baudry M Changes in polyamine levels and spectrin degradation following kainate-induced seizure activity: effect of difluoromethylornithine Exp Neurol 116 116 345 354 1587335 10.1016/0014-4886(92)90013-g

[B17] Katsifis A Mattner F Dikic B Papazian V Synthesis of substituted [123I]imidazo[1,2-a]pyridines as potential probes for the study of the peripheral benzodiazepine receptors using SPECT Radiochim Acta 2000 88 229 232 10.1524/ract.2000.88.3-4.229

[B18] Mattner F Katsifis A Staykova M Ballantyne P Willenborg DO Evaluation of a radiolabelled peripheral benzodiazepine receptor ligand in the central nervous system inflammation of experimental autoimmune encephalomyelitis: a possible probe for imaging multiple sclerosis Eur J Nucl Med Mol Imaging 2005 32 557 563 10.1007/s00259-004-1690-y 15875181

[B19] Bourdier T Pham TQ Henderson D Jackson T Lam P Izard M Katsifis A Automated radiosynthesis of [(18)F]PBR111 and [(18)F]PBR102 using the Tracerlab FX(FN) and Tracerlab MX(FDG) module for imaging the peripheral benzodiazepine receptor with PET Appl Radiat Isot 2012 70 1 176 183 10.1016/j.apradiso.2011.07.014 21852142

[B20] Katsifis A Loc'h C Henderson D Bourdier T Pham T Greguric I Lam P Callaghan P Mattner F Eberl S Fulham M A rapid solid-phase extraction method for measurement of non-metabolised peripheral benzodiazepine receptor ligands, [(18)F]PBR102 and [(18)F]PBR111, in rat and primate plasma Nucl Med Biol 2011 38 137 148 10.1016/j.nucmedbio.2010.07.008 21220137

[B21] Paxinos G Watson C The Rat Brain in Stereotaxic Coordinates 2007 6 Academic Press, London

[B22] Thompson MR Callaghan PD Hunt GE Cornish JL McGregor IS A role for oxytocin and 5-HT(1A) receptors in the prosocial effects of 3,4 methylenedioxymethamphetamine ("ecstasy") Neuroscience 2007 146 509 514 10.1016/j.neuroscience.2007.02.032 17383105

[B23] Mattner F Mardon K Katsifis A Pharmacological evaluation of [123I]-CLINDE: a radioiodinated imidazopyridine-3-acetamide for the study of peripheral benzodiazepine binding sites (PBBS) Eur J Nucl Med Mol Imaging 2008 35 779 789 10.1007/s00259-007-0645-5 18057934

[B24] Kemp BJ Hruska CB McFarland AR Lenox MW Lowe VJ NEMA NU 2–2007 performance measurements of the Siemens Inveon preclinical small animal PET system Phys Med Biol 2009 54 2359 2376 10.1088/0031-9155/54/8/007 19321924 PMC2888286

[B25] Van Vliet EA da Costa Araujo S Redeker S van Schaik R Aronica E Gorter JA Blood–brain barrier leakage may lead to progression of temporal lobe epilepsy Brain 2007 130 521 534 10.1093/brain/awl318 17124188

[B26] Zhang ZG Zhang L Tsang W Soltanian-Zadeh H Morris D Zhang R Goussev A Powers C Yeich T Chopp M Correlation of VEGF and angiopoietin expression with disruption of blood–brain barrier and angiogenesis after focal cerebral ischemia J Cereb Blood Flow Metab 2002 22 379 392 11919509 10.1097/00004647-200204000-00002

[B27] Nguyen VH Verdurand M Dedeurwaerdere S Wang H Zahra D Gregoire MC Zavitsanou K Increased brain metabolism after acute administration of the synthetic cannabinoid HU210: a small animal PET imaging study with 18 F-FDG Brain Res Bull 2012 87 172 179 10.1016/j.brainresbull.2011.11.011 22155282

[B28] Altar CA Baudry M Systemic injection of kainic acid: gliosis in olfactory and limbic brain regions quantified with [3 H]PK 11195 binding autoradiography Exp Neurol 1990 109 333 341 10.1016/S0014-4886(05)80024-X 2209775

[B29] Vezzani A Friedman A Brain inflammation as a biomarker in epilepsy Biomark Med 2011 5 607 614 10.2217/bmm.11.61 22003909 PMC3625731

[B30] Banati RB Goerres GW Myers R Gunn RN Turkheimer FE Kreutzberg GW Brooks DJ Jones T Duncan JS [11C](R)-PK11195 positron emission tomography imaging of activated microglia in vivo in Rasmussen's encephalitis Neurology 1999 53 2199 2203 10.1212/WNL.53.9.2199 10599809

[B31] Butler T Ichise M Teich AF Gerard E Osborne J French J Devinsky O Kuzniecky R Gilliam F Pervez F Provenzano F Goldsmith S Vallabhajosula S Stern E Silbersweig D Imaging inflammation in a patient with epilepsy due to focal cortical dysplasia J Neuroimaging 2011 10.1111/j.1552-6569.2010.00572.x PMC5303618 21223436

[B32] Owen DR Gunn RN Rabiner EA Bennacef I Fujita M Kreisl WC Innis RB Pike VW Reynolds R Matthews PM Parker CA Mixed-affinity binding in humans with 18-kDa translocator protein ligands J Nucl Med 2011 52 24 32 10.2967/jnumed.110.079459 21149489 PMC3161826

[B33] Owen DR Yeo AJ Gunn RN Song K Wadsworth G Lewis A Rhodes C Pulford DJ Bennacef I Parker CA StJean PL Cardon LR Mooser VE Matthews PM Rabiner EA Rubio JP An 18-kDa translocator protein (TSPO) polymorphism explains differences in binding affinity of the PET radioligand PBR28 J Cereb Blood Flow Metab 2012 32 1 5 10.1038/jcbfm.2011.147 22008728 PMC3323305

[B34] Tomkins O Shelef I Kaizerman I Eliushin A Afawi Z Misk A Gidon M Cohen A Zumsteg D Friedman A Blood–brain barrier disruption in post-traumatic epilepsy J Neurol Neurosurg Psychiatry 2008 79 774 777 10.1136/jnnp.2007.126425 17991703

[B35] Bottlaender M Valette H Delforge J Saba W Guenther I Curet O George P Dolle F Gregoire MC In vivo quantification of monoamine oxidase A in baboon brain: a PET study using [(11)C]befloxatone and the multi-injection approach J Cereb Blood Flow Metab 2010 30 792 800 10.1038/jcbfm.2009.242 19920845 PMC2949159

[B36] Delforge J Syrota A Bottlaender M Varastet M Loc'h C Bendriem B Crouzel C Brouillet E Maziere M Modeling analysis of [11C]flumazenil kinetics studied by PET: application to a critical study of the equilibrium approaches J Cereb Blood Flow Metab 1993 13 454 468 10.1038/jcbfm.1993.60 8478404

[B37] Reilac A Lehnert W Lin J Meikle SR Gregoire M-C Iterative-based partial volume effects correction with wavelet-based regularization for quantitative PET imaging IEEETrans Med Imaging Conf proc 2011 3788 3791

